# Pyoderma gangrenosum and spider bites: a case series

**DOI:** 10.1111/ddg.15731

**Published:** 2025-05-26

**Authors:** Luca Rapparini, Michelangelo La Placa, Giorgio De Benedetto, Yuri Merli, Annalucia Virdi, Cosimo Misciali, Federico Bardazzi

**Affiliations:** ^1^ Dermatology Unit IRCCS Azienda Ospedaliero‐Universitaria di Bologna Bologna Italy; ^2^ Department of Medical and Surgical Sciences Alma Mater Studiorum University of Bologna Bologna Italy

**Keywords:** Pyoderma gangrenosum, spider bite, ulcer

Dear Editors,

Pyoderma gangrenosum (PG) is a rare inflammatory skin disease classified in the group of neutrophilic dermatoses. Clinically, it is characterized by painful, irregular, erythematous‐purple skin ulcerations with undermined borders.^1^ The etiology of PG remains uncertain, and its pathogenesis is not yet fully understood. However, its frequent association with systemic diseases – such as chronic inflammatory bowel disease, rheumatoid arthritis, and hematological disorders – suggests an underlying immunological abnormality; nevertheless, 25–50% of cases are classified as idiopathic.^2^, ^3^ A characteristic feature of PG is the presence of the pathergy phenomenon, i.e. the appearance of new lesions or the worsening of pre‐existing ones as a result of minor trauma, surgical procedures, injections, or prick tests.^2^, ^4^ In this context, spider bites are interesting but little‐known trigger factors. Although there is a relatively large amount of literature on PG, few studies have investigated a possible etiological role.^3^, ^5^, ^6^

In Italy, spider bites are rare and usually cause localized erythema, edema, and itching or pain, which typically resolve within a few days without treatment. However, some dangerous species, including the Mediterranean black widow (*Latrodectus tredecimguttatus*) and the Mediterranean recluse spider (*Loxosceles rufescens*) can cause severe health issues.^7^

We collected five cases of PG that arose after spider bites in patients attending the Dermatology Unit of the University of Bologna from 2023 to 2024. Patients were informed about the use of their clinical information in accordance with the principles of the Declaration of Helsinki and about the use of photos for publication purposes. All patients presented ulcerative lesions that initially simulated necrotizing bacterial infections, and they had been treated with antibiotics (amoxicillin/clavulanate or azithromycin) and antiseptics such as iodopovidone. However, in all patients, the treatment had been ineffective, confirming the diagnosis of PG. The patients showed ulcers ranging in size from 1.5 to 15 cm, often with irregular margins and fibrin and hypertrophic bottom. The surrounding skin was frequently erythematous or purpuric (Figure 1a–e). The localization was predominantly in the lower extremities, with one abdominal case. The diagnosis was supported by histological examinations (Figure 1f). The treatments set were mainly topical clobetasol propionate under occlusion. In some cases, the addition of systemic immunosuppressants such as cyclosporine or methylprednisolone was necessary. Healing times ranged from 6 to 22 weeks, with residual hyperchromic outcomes. The clinical characteristics are summarized in Table 1.

**FIGURE 1:**
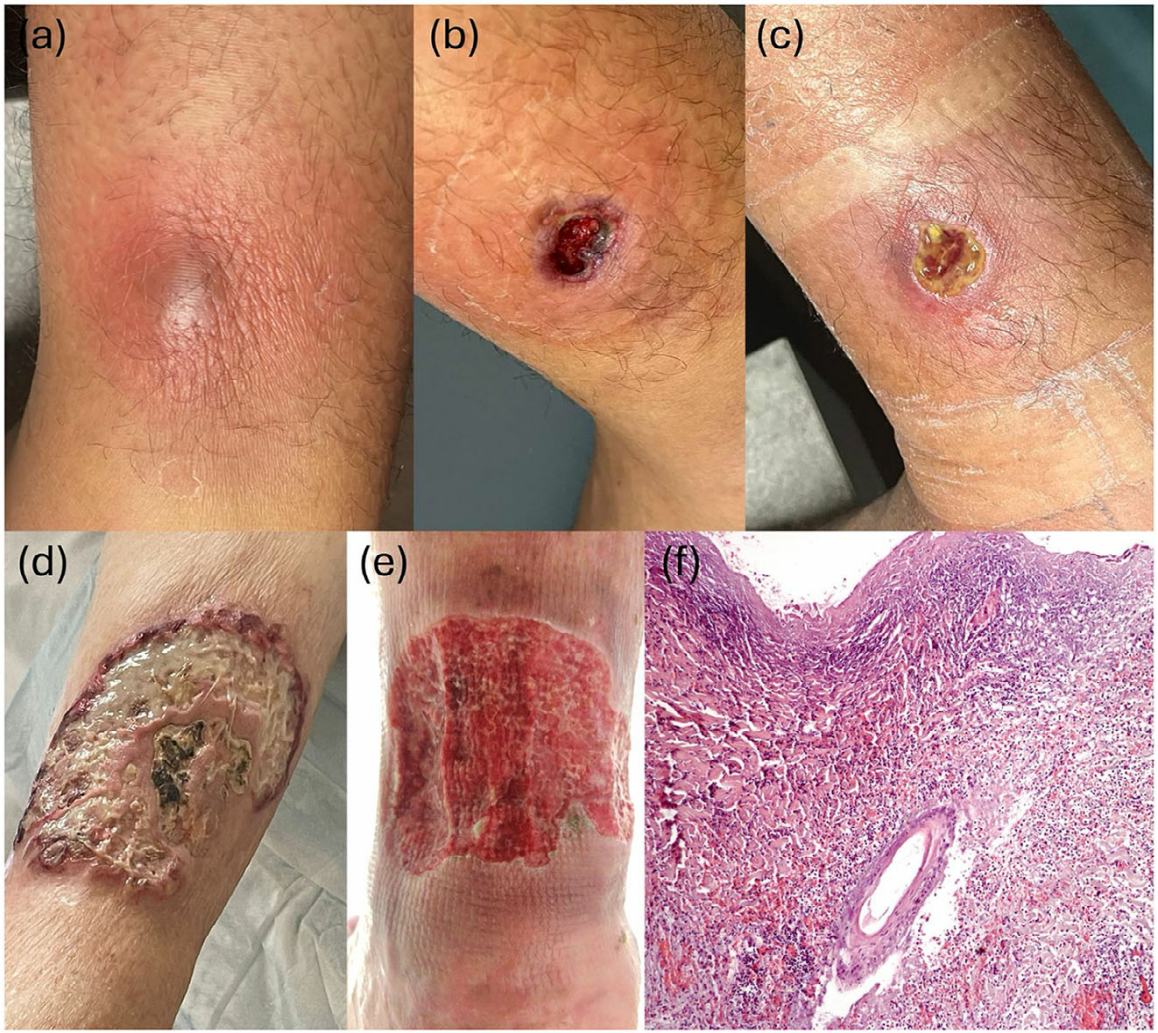
Evolution of the lesion in patient 1. (a) Four days after the spider bite. (b) Pyoderma gangrenosum (PG) diagnosis on day 13. (c) Progressive healing of the lesion on day 30. (d) Presentation at PG diagnosis of patient 2 and (e) patient 4. (f) Histological examination of patient 2, showing a dense and diffuse infiltrate of neutrophils extending throughout the dermis, degeneration of collagen in the dermis, folliculitis and perifolliculitis, and abscess formation with rupture of the follicular infundibulum (hematoxylin‐eosin stain, original magnification x 10).

**TABLE 1:** Characteristics of patients with pyoderma gangrenosum occurring after a spider bite.

Pt.	Sex, age (years)	Previous comorbidities	Characteristics of the lesion	Treatment set after spider bite	Occurrence of PG after spider bite (days)	Treatment set for PG	PG healing time (days)
1	M, 33	Cutaneous psoriasis in brodalumab therapy	Circular ulcer, 2 cm in diameter, with a hypertrophic base and fibrous coating, located on the lateral side of the left leg.	Azithromycin 500 mg, for 3 days Fusidic acid + betamethasone cream, once daily	13	Clobetasol propionate cream under occlusion, once daily	70
2	F, 84	Arterial hypertension, previous myocardial infarction, diverticulosis	Ulcer measuring 15 × 10 cm, with a fibrinous and granulating base, small necrotic eschar, and erythematous‐purplish perilesional skin, located on the right leg	Amoxicillin/clavulanic acid 875/125 mg capsules, three times daily for 14 days	20	Clobetasol propionate cream under occlusion, once daily	82
3	F, 53	Hashimoto's thyroiditis in replacement therapy, seronegative arthritis under NSAIDs, fibromyalgia	Plaque with a maximum diameter of 7.5 cm, featuring multiple ulcerations and indistinct margins, surrounded by erythematous‐purplish perilesional skin; located on the lateral supramalleolar region of the left leg	Amoxicillin/clavulanic acid 875/125 mg capsules, three times daily for 8 days	32	Methylprednisolone 16 mg capsules: 2 capsules daily for 7 days, then 1.5 capsules daily for 7 days, then 1 capsule daily for 7 days, then 0.5 capsule daily for 1 month, followed by 0.25 capsule daily for 1 month	62
4	M, 55	Mild cutaneous psoriasis in topical therapy	Ulcer of irregular oval shape, 5 cm in diameter, with a hypertrophic base and fibrous coating, located on the posterior aspect of the left ankle	Iodopovidone gauze + elastocompression dressing	25	Clobetasol propionate ointment under occlusion, applied once daily Cyclosporine 100 mg capsules, taken twice daily	160
5	M, 24	None	Oval‐shaped ulcer, 1.5 cm in diameter, with a hypertrophic base, located on the right side of the abdomen	Iodopovidone gauze	37	Clobetasol propionate cream under occlusion, once daily	40

*Abbr*.: PG, pyoderma gangrenosum

Pyoderma gangrenosum is a frequently misdiagnosed condition, particularly in its initial phases. In our experience, patients were initially treated for bacterial infections. This diagnostic delay highlights the need for greater awareness by physicians in considering PG in the differential diagnosis of ulcerative lesions not responsive to conventional therapy.^4^, ^8^

The possible link between spider bites and PG raises important questions: local trauma and acute inflammation from venom could trigger an aberrant immune response (pathergy phenomenon). Trauma is a well‐established stimulus for the release of cytokines and damage‐associated molecular patterns that potentiate innate immune responses. Trauma has been shown to induce the release of interleukin (IL)‐8 and IL‐36 from keratinocytes, both of which are putative PG‐driving cytokines. Tissue injury can also promote release of autoantigens. These processes may be sufficient to trigger PG, especially in individuals with pathogenic variants in inflammasome‐related genes.^9^ It can be hypothesized that minor trauma from the spider bite, combined with the release of toxins and enzymes in the spider venom, triggers the activation of the same cascade. It would be interesting to investigate whether there are toxins or proteins in spider venom that can amplify inflammatory responses in genetically or immunologically predisposed individuals.

In conclusion, we believe that various potential causes should be considered in cases of PG, including spider bites as a rare but possible triggering factor.^10^

## CONFLICT OF INTEREST STATEMENT

None.
